# Therapy of the patients with HIV/TB infection and dynamics of level 'naïve' CD4-lymphocytes

**DOI:** 10.1186/1758-2652-13-S4-P196

**Published:** 2010-11-08

**Authors:** AA Popova, AV Kravchenko, GM Kozhevnikova, LV Serebrovskaya

**Affiliations:** 1 Peoples’ Friendship University of Russia, Moscow, Russian Federation; 2 Russian Federal AIDS Center, Moscow, Russian Federation

## Background

Increase of number of patients with HIV/TB and prevalence of the given pathology in structure of the reasons of death rate of HIV-infected patients.

## Objective

Study of indicators of cellular immunity and their change in the course of therapy at patients with HIV/TB.

## Methods

106 patients were studied in 4 groups: 39 HIV/TB co-infected individuals (HIV+/TB+), 25 patients with HIV infection, 17 HIV-negative patients with active pulmonary TB (HIV-/TB+) and 25 healthy controls. Measures of T-cells and viral load were at baseline and after initiation of HAART and/or antitubercular therapy (4 and 12 weeks) for potential immune correlates of disease progression and prognosis. Definition immune indicators were spent by flow cytometry (BD Biosciences, USA).

## Results

**Figure 1:**
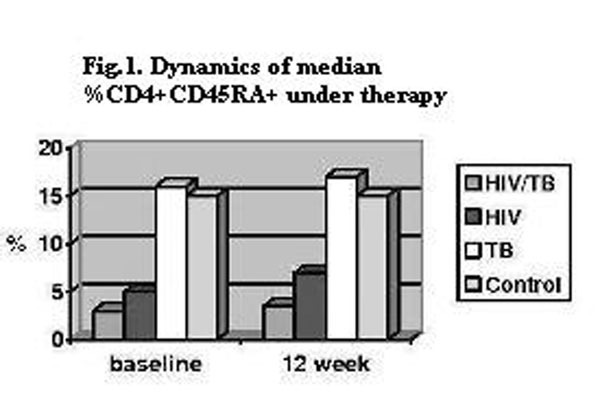


Before treatment: percent of CD4+CD45RA+ differs in investigated groups. The lowest values registered at patients of 1 and 2 groups (fig.1). For patients in the group 3, the deviation of the given indicator from control group was small. If HAART and antitubercular therapy were effective we registered increase level % CD4+CD45RA+ in all groups. ∆ % CD4+CD45RA+ increased more slowly in patients of group 1. The same results turn out at research of absolute number CD4+CD45RA+ in all groups.

## Conclusions

Low-level % CD4+CD45RA+ in patients with HIV/TB and HIV infection is connected with the general decrease ÑD4-lymphocytes. After first months of efficient therapy of patients with HIV/TB, infringements in immune system still remained, which, probably, are caused by joining of opportunistic infections.

